# Comparing Levels of Positive Mental Well‐Being and Life Satisfaction Between Mothers and Fathers of Children With an Intellectual Disability

**DOI:** 10.1111/jir.70021

**Published:** 2025-07-26

**Authors:** Marina Antoniadou, Vaso Totsika, Emma Langley, Richard P. Hastings, Caitlin A. Williams

**Affiliations:** ^1^ Division of Psychiatry University College London London UK; ^2^ Tavistock & Portman NHS Foundation Trust London UK; ^3^ Education Studies University of Warwick Coventry UK; ^4^ Intellectual Disabilities Research Institute (IDRIS) University of Birmingham Birmingham UK; ^5^ School of Psychology University of Birmingham Birmingham UK

**Keywords:** intellectual disability, life satisfaction, mental well‐being, positive mental health, SWEMWBS

## Abstract

**Background:**

Positive mental health is experienced by parents of children with intellectual disability and has been shown to be associated with child mental health outcomes in these families. There is a dearth of evidence on mother–father differences in levels of positive mental health, despite evidence of differences in levels of mental health problems. This study aimed to compare levels of positive mental health, namely life satisfaction and mental well‐being, between mothers and fathers of children with an intellectual disability.

**Method:**

Participants were 85 mother–father dyads participating in the 1000 Families Study, a UK‐based cohort study of families of children with intellectual disability. Approximately 92% of the dyads were a couple, while the rest reported they were divorced, separated or not living with the child's other parent. Over 90% of participants were the child's biological parent. The mean age of mothers and fathers was 45 years old and 50.5 years old, respectively. Children were on average 14.8 years old (ages ranged from 9 to 20 years old) and over 70% were boys.

**Results:**

Multilevel models initially showed no differences between mothers' and fathers' mental well‐being and life satisfaction. After controlling for other factors potentially related to positive mental health, findings indicated that life satisfaction was reported at higher levels by mothers compared with fathers of children with intellectual disability.

**Conclusions:**

Subject to replication in future studies, initial evidence of gender differences in life satisfaction highlights that fathers of children with an intellectual disability may be particularly vulnerable to lower levels of positive mental health. Fathers may require additional support for achieving positive mental health.

Research in the intellectual disability field has predominantly focused on the ‘negative narrative’ surrounding parents of children with intellectual disability (Hastings 2016), viewing the child with intellectual disability as having an overall negative impact on parents' mental health. Nevertheless, parents often report positive experiences related to raising a child with intellectual disability at the same time as negative experiences, including improved family relationships and increased parental positivity (Jess et al. 2018).

Positive mental health refers to a condition of well‐being in which the person is aware of their abilities, can manage life's regular stresses, work effectively and efficiently and positively contribute to their community (WHO 2022). Therefore, positive mental health is a multidimensional construct that includes both mental well‐being and life satisfaction. Mental well‐being encompasses both hedonic (e.g., happiness and the presence of pleasure) and eudaimonic (e.g., self‐realisation and purpose) aspects of well‐being (Thrash 2021). Life satisfaction is considered under hedonic well‐being, describing an individual's cognitive evaluation of their own life (Diener 1984).

Positive mental health does not simply represent the absence of mental health problems. Therefore, this is a distinct construct that warrants separate consideration (Keyes 2002). Parental positive mental well‐being has been positively associated with child well‐being (Newland 2015). However, positive mental health in parents of children with intellectual disability has received little research attention.

In a population‐representative study of mothers of children with intellectual disability, levels of life satisfaction were similar to those of mothers of children without intellectual disability (Totsika et al. 2011a) though a more recent meta‐analysis suggested that life satisfaction in mothers of children with Down syndrome may be lower than that of mothers of typically developing children (Rutter et al. 2024). Studies on life satisfaction of fathers have found lower levels of life satisfaction in fathers of children with intellectual disability compared with fathers of children without intellectual disability (Langley et al. 2020) and similar levels to the life satisfaction of fathers of autistic children (Gugliandolo et al. 2022). As evident, studies on positive mental health of mothers and fathers of children with intellectual disability are limited, and there is a dearth of evidence on mother–father differences in positive mental health (Dunn et al. 2019; Rutter et al. 2024).

Studies outside of the intellectual disability field have found that, compared with men, women tend to report lower levels of mental well‐being (Koushede et al. 2019; Tennant et al. 2007), but higher levels of life satisfaction (Blanchflower and Bryson 2024; Blanchflower and Oswald 2004). Becchetti and Conzo (2022) explain these findings through the affect intensity rationale, describing women as being affected more by both positive and negative events in life. However, Blanchflower and Bryson (2024) hypothesise that these gender differences are potentially influenced by social and structural factors, such as unequal access to resources, differences in caregiving expectations and gender inequality. At the same time, research on negative mental health in parents of children with an intellectual disability tends to find differences between mothers and fathers, with mothers generally reporting worse levels of negative mental health (Dunn et al. 2019). These differences could potentially be attributed to differences in parenting roles that can remain conflated with gender.

Given that higher levels of positive mental health in parents of children with intellectual disability are associated with lower levels of children's behavioural and emotional problems (Langley et al. 2020; Totsika et al. 2011b), it is important that research examines potential differences in positive mental health levels between mothers and fathers of children with an intellectual disability. This is an important first step towards building a more complete picture of the mental health of parents of children with an intellectual disability and could inform the development of more tailored and targeted family support services for parents who may be at risk of reduced well‐being.

To examine potential differences in the positive mental health of mothers and fathers, research needs to control for factors known to be associated with positive mental health. However, there is a lack of theoretical models of positive mental health, both in parents and in the general population, to help guide the selection of confounders. Child emotional and behavioural problems, child age and family income are variables that have been associated with life satisfaction and mental well‐being in mothers and fathers of children with intellectual disability (Langley et al. 2020; Park and Kim 2019; Totsika et al. 2011a; Totsika et al. 2011b) or autistic children/children with developmental disabilities (Budler et al. 2023; Cho and Kahng 2014; Landon et al. 2017; Salas et al. 2017; Salomone et al. 2017). These factors (i.e., child age, child emotional and behavioural problems and family income) have also demonstrated associations with negative mental health, and research has also suggested associations with marital relationship satisfaction and relationships with grandparents (Dunn et al. 2019; Hastings et al. 2002; Heller et al. 2000; Kersh et al. 2006; Langley et al. 2017; Trute 2003). Therefore, these are all factors that may affect the positive mental health of parents of children with an intellectual disability and need to be controlled for when examining differences between mothers and fathers.

The present study aimed to compare levels of positive mental health, namely life satisfaction and mental well‐being, between mothers and fathers of children with an intellectual disability. We drew on data from a large United Kingdom (UK) cohort of families of children with an intellectual disability from the 1000 Families Study, where data from fathers were collected at one of the waves to enable a comprehensive investigation of families of children with an intellectual disability. Given the limited research on differences in positive mental health in mothers and fathers of children with intellectual disabilities, we did not formulate directional hypotheses. Instead, this study takes an exploratory approach to identify potential differences in parental positive mental health and its associated factors.

## 1 Methods

### 1.1 Participants

Participants consisted of 85 mother–father dyads of children and adolescents with intellectual disability aged between 9 and 20 years who took part in Wave 3 of the 1000 Families Study (Hastings et al. 2020). Parent respondents were mostly biological mothers (*n* = 80) and biological fathers (*n* = 77). Grandparents who reported being the primary caregivers of children were also included in the study (*n* = 2).

Most mothers (*n* = 74) and fathers (*n* = 74) described themselves as White British. The majority of participants were married and/or living with their partner (*n* = 74). The remaining parents reported they were divorced, separated or not living with their partner (*n* = 10). Dyads were assumed to be couples if both parents reported being married and/or living with their partner and provided the same postcode. Based on these criteria, most dyads (*n* = 78) were identified as couples living in the same home. Of the remaining dyads, only one father described himself as married and living with a partner, while the others were divorced, separated or not living with their partner.

Around half of the mothers (*n* = 45) and fathers (*n* = 38) were educated to a university degree level. Tables 1 and 2 summarise the demographic characteristics of fathers and mothers respectively. Table 3 summarises the descriptive statistics for the children and adolescents with intellectual disability (*n* = 85).

**TABLE 1:** Father demographic information (*n* = 85).

Variables	
**Mean age (SD)**	50.5 (8.1)
**Relationship to child (%)**	
Biological father	77 (90.6%)
Adoptive father	3 (3.5%)
Stepfather	2 (2.4%)
Foster father	1 (1.2%)
Grandfather	2 (2.4%)
**Ethnicity (%)**	
White British	74 (87.1%)
White other (Irish, travelling community, other)	6 (7.0%)
Other ethnic background	5 (6.0%)
**Parent couples in the same household (%)** ^**a**^	78 (91.8%)
**Marital status (%)**	
Married and living with spouse/civil partner	69 (81.2%)
Living with partner	5 (5.9%)
Divorced/separated/single/not currently living with partner	10 (11.8%)
Missing information	1 (1.2%)
**Employment status (%)** ^**b**^	
In a job and currently working for an employer	60 (70.6%)
Self‐employed	13 (15.3%)
Doing voluntary work	1 (1.2%)
Carer for their child with intellectual disability	6 (7.1%)
Carer for somebody else	2 (2.4%)
Looking after home and family	2 (2.4%)
Doing something else	8 (9.4%)
**Qualifications (%)**	
Degree level (e.g., BA, BSc, MA)	38 (44.7%)
Higher education but below degree level	14 (16.5%)
A/AS levels or equivalent	7 (8.2%)
5 or more GCSEs at A*–C or equivalent	7 (8.2%)
Some GCSEs passes or equivalent	15 (17.6%)
No educational qualifications	4 (4.7%)
**Relative income poverty (%)**	
Above median weekly household income (more than £700)	38 (44.7%)
Below median weekly household income (less than £700)	46 (54.1%)
Missing information	1 (1.2%)

^a^ Parent couples in the same household were defined as mother–father dyads who reported being married or living with their partner and providing the same postcode.

^b^ Carers were asked to select all occupation options that applied to them.

Abbreviation: SD, standard deviation.

**TABLE 2:** Mother demographic information (*n* = 85).

Variables	
**Mean age (SD)**	45 (6.5)
**Relationship to child (%)**	
Biological mother	80 (94.0%)
Adoptive mother	3 (3.6%)
Grandmother	2 (2.4%)
**Ethnicity (%)**	
White British	74 (87.1%)
White other (Irish, other)	6 (7.1%)
Other ethnic background	4 (4.8%)
Missing information	1 (1.2%)
**Marital status (%)**	
Married and living with spouse/civil partner	69 (81.2%)
Living with partner	5 (5.9%)
Divorced/separated/single/not currently living with partner	10 (11.8%)
Missing information	1 (1.2%)
**Employment status (%)** ^**a**^	
In a job and currently working for an employer	40 (47.1%)
Self‐employed	12 (14.1%)
Doing voluntary work	8 (9.4%)
Carer for their child with intellectual disability	47 (55.3%)
Carer for somebody else	9 (10.6%)
Looking after home and family	33 (38.8%)
Full‐time student	2 (2.4%)
Doing something else	3 (3.5%)
Unemployed	2 (2.4%)
**Qualifications (%)**	
Degree level (e.g., BA, BSc, MA)	45 (52.9%)
Higher education but below degree level	16 (18.8%)
A/AS levels or equivalent	13 (15.3%)
5 or more GCSEs at A*–C or equivalent	2 (2.4%)
Some GCSEs passes or equivalent	7 (8.2%)
No qualifications	1 (1.2%)
Missing information	1 (1.2%)
**Relative income poverty (%)**	
Above median weekly household income (more than £700)	39 (45.9%)
Below median weekly household income (less than £700)	44 (51.8%)
Missing information	2 (2.4%)

*Note:* Mothers were not asked to answer questions regarding time‐invariant variables at Wave 3 and so ethnicity data were taken from Wave 1 of the 1000 Families Study.

^a^ Carers were asked to select all occupation options that applied to them.

Abbreviation: SD, standard deviation.

**TABLE 3:** Demographic information of the children and adolescents with intellectual disability (*n* = 85).

Variables	
**Mean age (SD)**	14.8 (2.7)
**Gender (%)**	
Male	60 (70.6%)
Female	24 (28.2%)
Missing information	1 (1.2%)
**Additional diagnoses (%)**	
Autism	36 (42.2%)
Down syndrome	10 (11.8%)

Abbreviation: SD, standard deviation.

### 1.2 Measures

#### 1.2.1 Positive Mental Well‐Being

Mothers and fathers completed the Short Warwick‐Edinburgh Mental Well‐being Scale (SWEMWBS; Stewart‐Brown et al. 2009), a measure of positive mental well‐being. The SWEMWBS is a seven‐item scale assessing both hedonic and eudaemonic aspects of well‐being (Marmara et al. 2022) with positively worded items (e.g., ‘I have been feeling useful’). Items are rated on a five‐point Likert scale ranging from one (none of the time) to five (all of the time). Higher scores indicate higher levels of mental well‐being. Raw total scores were transformed to metric scores, allowing for better interpretation of findings (Warwick Medical School 2023). Scores were classified as low (0–20), moderate (20–30) and high (> 30), as suggested by previous studies (Santini et al. 2022). Cronbach's alpha was 0.87 for mothers and 0.88 for fathers, indicating good internal consistency (Tavakol and Dennick 2011).

#### 1.2.2 Life Satisfaction

Life satisfaction was assessed using a single item asking mothers and fathers ‘how satisfied are you with the way your life has turned out so far’ (Department of Environment, Food and Rural Affairs 2011). Participants indicated their response to this statement on a scale of one (completely dissatisfied) to 10 (completely satisfied). Scores were categorised as low (0–4), moderate (5–6), high (7–8) and very high (9–10) (Office of National Statistics 2022). The single‐item life satisfaction scale demonstrated high criterion validity with established life satisfaction measures including several items (Cheung and Lucas 2014). The single‐item life satisfaction scale has been used in studies of parents of children with intellectual disability (e.g., Langley et al. 2020).

#### 1.2.3 Child Behavioural and Emotional Problems

The Strengths and Difficulties Questionnaire (SDQ; Goodman 1997), assessing the behavioural and emotional well‐being of a child with intellectual disability, was completed by mothers. The SDQ is a 25‐item questionnaire, comprising five subscales: emotional symptoms, conduct problems, hyperactivity/inattention problems, peer relationship problems and prosocial behaviour. Items are rated on a three‐point scale from zero (not true) to two (certainly true). A total score of difficulties was created by adding the scores of the first four subscales together. The SDQ accurately assesses the behavioural and emotional problems of children with intellectual disability (Murray et al. 2020). Cronbach's alpha for the total difficulties of children in this sample was 0.75, suggesting an acceptable level of internal consistency (Tavakol and Dennick 2011). Wave 3 of the 1000 Families Study did not measure the children's behavioural and emotional problems for families of children over the age of 16 in part for consent reasons, as parents had to report data about the young person, and in part as the SDQ is not usually used beyond age 16. Therefore, this information was drawn from the second wave.

#### 1.2.4 Interparental Relationship Satisfaction

Mothers and fathers answered a single item assessing interparental relationship satisfaction. Parents rated how happy or unhappy they are in their relationship with their partner on a seven‐point Likert scale of one (very unhappy) to seven (very happy). The same method was used to measure relationship satisfaction in previous studies including the Millennium Cohort Study (University of London 2007).

#### 1.2.5 Grandparental Support and Conflict

The mother's and father's perceived support and conflict with the child's grandparents were assessed using the four‐item grandparent support–conflict measure (Hastings et al. 2002). Items were rated on a four‐point scale from *strongly disagree* to *strongly agree*. One item measured practical support (‘my parents are very much involved in the practical aspects of bringing up my child with intellectual disability’) and one item measured emotional support (‘my parents are a good source of emotional support for me’). Conflict with grandparents was measured by the remaining two items (e.g., ‘my parents and I disagree over the care of my child with intellectual disability’). Weighted mean scores were computed for support and conflict subscales with grandparents. Higher scores in the support items indicated higher support from grandparents. The items measuring conflict were reversed scored so that higher ratings indicated less conflict. Internal consistency in this sample was considered acceptable (Tavakol and Dennick 2011; Cronbach's *α*: 0.79 for fathers and 0.75 for mothers).

#### 1.2.6 Family Poverty

A poverty composite variable was created for both mothers and fathers based on their individual responses to three items measuring income poverty, subjective poverty and hardship. Parents reported their total weekly family income after any deductions, which was used to determine if their income was above or below the median in the UK. They also rated their financial situation on a five‐point scale ranging from *living uncomfortably* to *finding it very difficult*. Hardship was measured by asking how difficult it would be to raise £2000 in an emergency, with responses ranging from *I could easily raise the money* to *I don't think I could easily raise the money*. Higher composite scores indicated higher individual poverty levels.

### 1.3 Procedure

This study used survey data from Waves 2 and 3 of the 1000 Families Study, a longitudinal cohort study collecting data from parents of children with intellectual disability living in the UK (Hastings et al. 2020). Participants were initially recruited through a project website, social media, charity and organisation newsletters and through communications with special schools and parent support organisations across the UK. At Wave 1, inclusion criteria required families to live within the UK and have one or more children aged between 4 years and 15 years 11 months with an intellectual disability. Inclusion criteria also required parents to be over 18 years old and have sufficient English skills to be able to independently complete the survey. The children with intellectual disabilities needed to be living in the family home and intellectual disability diagnoses were confirmed via parental report.

Wave 2 data collection took place from August 2018 until January 2021 while Wave 3 began in December 2021 and continued until April 2023. Ethical approval was obtained by the University of Warwick Humanities and Social Sciences Research Ethics Committee (HSSREC) (reference number: HSSREC 197/20‐21 AM02) and participants provided informed consent. Parents who participated in Wave 1 or 2 of the study received an email link to complete the Wave 3 questionnaire. Mothers who participated in Wave 3 (who had consented to be contacted for future research) were then emailed an invitation to share with the fathers or male carers of their child. Each participant was offered a £10 voucher as appreciation for their time.

### 1.4 Statistical Analysis

To examine initial differences between mothers' and fathers' mental well‐being and life satisfaction, paired samples t‐tests were conducted. Multilevel models (MLM) were fitted to compare mother–father differences while controlling for factors known to be associated with well‐being, providing a way of adjusting the mother–father comparison. MLM clusters individuals into higher‐order groups and accounts for interdependence in their analyses (Heck et al. 2013). Therefore, MLM accounted for the nested nature of the data, where parents were clustered within the same household. Separate MLMs were conducted for life satisfaction and mental well‐being outcomes.

Confounders in the MLM models were selected on the basis of factors shown to be associated with mental health outcomes in previous studies and availability within the database, given the absence of theoretical models of positive well‐being. Data were structured in a two‐level hierarchical model with Level 1 including variables reported separately by each parent, and Level 2 including variables measured once per dyad. Level 1 variables included parent gender, relationship satisfaction, family poverty and grandparent support and conflict, reported individually by mothers and fathers. Level 2 variables included behavioural and emotional problems of the child with intellectual disability, and the child's age measured at the family level. Family was modelled as a random factor while the rest of the variables were modelled as fixed factors.

To assess the model's parameters, the variance components (VC) covariance structure was employed, which generates a different variance estimate for every random effect (Heck et al. 2013). Across all variables, the percentage of missing values ranged from 0.6% (level of poverty) to 27.3% (grandparental conflict). Because the amount of missing data was relatively high (> 10%), Little's MCAR test (Little 1988) was conducted to test if the data were missing completely at random (MCAR). The test was not significant (*p* = 0.201), suggesting that the data were MCAR. Additionally, correlation coefficients were examined between missingness in predictor variables and observed outcomes. The results revealed low correlation coefficients, ranging from 0.018 to 0.132. Therefore, considering the low correlation coefficients and Little's MCAR test, there was no evidence that data were not missing at random and Maximum Likelihood (ML; Arbuckle 1996) was a suitable estimator for the models. ML assumes that the data are at least missing at random (MAR) and handles missing data by identifying the population parameter values that most probably generated the data being observed (Peugh and Enders 2004). Statistical analyses were conducted in SPSS Statistics, Version 28.0.

## 2 Results

Paired samples *t*‐tests revealed no statistically significant differences between the mental well‐being scores of fathers (M = 21.95, SD = 4.39) and mothers (M = 21.79, SD = 4.97) of children with intellectual disability (*t*(82) = 0.043, *p* = 0.966), with both parents reporting similar SWEMWBS scores. Similarly, there were no statistically significant differences in life satisfaction mean scores between fathers (M = 6.47, SD = 2.12) and mothers (M = 6.43, SD = 2.09); *t*(81) = 0.093, *p* = 0.926. Descriptive statistics for all variables and correlations between mothers' and fathers' individual reports are presented in Table 4.

**TABLE 4:** Descriptive statistics and correlations between mother and father reports.

	Mothers	Fathers	Total	Correlations
Variable	M (SD)	M (SD)	M (SD)	Pearson's *r*
Life satisfaction	6.43 (2.09)	6.47 (2.12)	6.45 (2.10)	0.321**
Mental well‐being	21.79 (4.97)	21.95 (4.39)	21.87 (4.67)	0.328**
Relationship satisfaction	5.47 (1.57)	5.69 (1.50)	5.59 (1.53)	0.655**
Poverty	3.67 (0.79)	3.57 (0.83)	3.62 (0.80)	0.331**
Grandparental support	1.84 (0.82)	2.06 (0.83)	1.93 (0.83)	0.097
Grandparental conflict	2.78 (0.76)	2.51 (0.92)	2.63 (0.86)	0.119
SDQ child with ID	18.90 (6.41)	—	18.90 (6.41)	—

*Note: r =* Pearson correlation between mothers' and fathers' individual report; **p < 0.01.

As a first step to fitting the MLMs, null models (with no predictors) were fitted to estimate the proportion of variance in life satisfaction and mental well‐being that lies within (Level 1) and between families (Level 2). The results of the mental well‐being null model in the current sample suggested that a MLM was justified. Intercepts varied significantly between families (Wald *Z* = 2.477, *p* = 0.013), with the ICC (0.29) suggesting that around 29% of the overall variability in mental well‐being scores was present across families. Similarly, intercepts varied significantly across families in the life satisfaction null model (Wald *Z* = 2.981, *p* = 0.003) with the ICC value being 0.36 (36%).

### 2.1 Individual‐Level (Level‐1) Random Intercept Models

When introducing Level 1 fixed factors in the models, parent gender was not associated with mental well‐being (*β* = 0.236, *p* = 0.745). Of the Level 1 confounders, relationship satisfaction was significantly associated with mental well‐being (*β* = 0.872, *p* = 0.004).

In the model examining Level 1 predictors of life satisfaction, parent gender was significantly associated with life satisfaction; mothers reported significantly higher life satisfaction scores than fathers within families (*β* = 1.07, *p* = 0.002). Of the Level 1 confounders, relationship satisfaction was associated with life satisfaction (*β* = 0.725, *p* < 0.001).

### 2.2 Group‐Level (Level 2) Random Intercept Models

Adding Level 2 predictors in the final model of well‐being accounted for a shift in the variance of SWEMWBS scores of 5.71% within families and 60.95% between families. In the final model of mental well‐being, parent gender was not significantly associated with mental well‐being (*β* = 0.526, *p* = 0.493); mothers and fathers scored similarly in the mental well‐being measure. Of the Level 1 and Level 2 confounders, interparental relationship satisfaction was significantly associated with mental well‐being (*β* = 0.801, *p* = 0.011).

In the final model of life satisfaction, the addition of all the predictors accounted for 22.19% of the life satisfaction score variance within families and 26.11% between families. Parent gender was a significant predictor of life satisfaction, with mothers reporting significantly higher life satisfaction scores than fathers (*β* = 1.179, *p* = 0.002). Of the Level 1 and Level 2 confounders, relationship satisfaction was significantly associated with life satisfaction scores (*β* = 0.704, *p* < 0.001). Table 5 presents the results of the final models of mental well‐being and life satisfaction outcomes.

**TABLE 5:** Final multilevel model results for mental well‐being and life satisfaction.

	Mental well‐being				Life satisfaction			
Variable	*B*	SE	*p*	95% CI	*B*	SE	*p*	95% CI
Parent gender	0.526	0.760	0.493	[−1.013, 2.065]	1.179	0.352	0.002	[0.468, 1.889]
*Controlling for*								
Relationship satisfaction	0.801	0.303	0.011	[0.192, 1.409]	0.704	0.156	< 0.001	[0.392, 1.015]
Poverty	−0.884	0.496	0.079	[−1.873, 0.105]	−0.438	0.250	0.084	[−0.937, 0.061]
Grandparental support	−0.034	0.482	0.944	[−0.995, 0.928]	0.223	0.247	0.370	[−0.270, 0.716]
Grandparental conflict	−0.35	0.447	0.937	[−0.927, 0.856]	0.100	0.227	0.662	[−0.354, 0.553]
Age child with ID	−0.007	0.186	0.970	[−0.385, 0.371]	0.057	0.106	0.596	[−0.158, 0.271]
SDQ child with ID	−0.087	0.064	0.182	[−0.217, 0.043]	−0.054	0.036	0.149	[−1.270, 0.020]

Abbreviations: CI, confidence interval; SDQ, Strengths and Difficulties Questionnaire; SE, standard error.

## 3 Discussion

The present study explored mother–father differences in life satisfaction and mental well‐being in families of children with an intellectual disability. After adjusting for factors associated with positive mental health, a significant gender difference emerged for life satisfaction; mothers reported higher life satisfaction scores compared with fathers. While it is likely that mothers and fathers may have interpreted the life satisfaction item differently, there is currently no evidence of measurement invariance or, conversely, gender bias for this measure. This difference aligns with UK population evidence that life satisfaction in women is higher compared with men, after controlling for factors often associated with life satisfaction, such as income and marital status (Blanchflower and Oswald 2004). No previous studies have compared life satisfaction between mothers and fathers of children with intellectual disability. An older, small study of parents of children with Down syndrome suggested levels of high satisfaction appeared similar between mothers and fathers, though no statistical comparison was attempted (Bränholm and Degerman 1992).

In the present study, no gender differences were evident for mental well‐being. This was the first study to measure mental well‐being using the short version of the WEMWBS (SWEMWBS; Stewart‐Brown et al. 2009) in parents of children with intellectual disability. Whole population studies have shown gender differences in the WEMWBS, with men scoring significantly (but only slightly) higher than women, but find no gender differences on the SWEMWBS (Koushede et al. 2019). This difference may be related to how certain items of the measure perform across men and women. For example, Marmara et al. (2022) found women scored lower than men on the WEMWBS item ‘I've got energy to spare’, potentially suggesting that experiences typically related to gender, such as increased caregiving demands and having less financial stability, may lead to women having less energy to spare. Additionally, interparental relationship satisfaction was significantly and positively associated with both mental well‐being and life satisfaction. This aligns with previous evidence that increased relationship quality predicted less parenting stress and depression over other factors such as socioeconomic status and social support (Kersh et al. 2006; Langley et al. 2017).

As this is the first study to compare positive mental health between mothers and fathers of children with intellectual disability, it is not clear whether the pattern of findings in the present study reflects gender differences or differences in the parenting role: 55.3% of mothers reported being the primary carer for their child with intellectual disability compared with just 7.1% of fathers. Mothers may spend more time with their child, which could lead to more opportunities for positive interactions and increased involvement in caregiving. This could also influence how mothers perceive their relationship with their child (Vilaseca et al. 2020; Zabidi et al. 2023). Although this study did not measure the amount of time each parent spent with their child, the hypothesis above may be supported by reported differences in employment, with 70.6% of fathers reporting being in employment compared with 47.1% of mothers. Differences in employment are likely to influence caregiving roles and mental health outcomes. Investigating differences between outcomes for mothers and fathers is conflated with both gender and caregiving responsibilities, meaning we cannot dissociate these two concepts. We have taken several intersectional characteristics into account while exploring factors that may be associated with parental well‐being, such as gender and socioeconomic status. However, future research should also account for other variables, such as perceptions of caregiving responsibilities, and how these might impact positive mental health.

To our knowledge, this is the first study comparing positive mental well‐being and life satisfaction outcomes in mothers and fathers of children with intellectual disability, adding to the limited existing research in this field. Nevertheless, limitations must also be considered. This was a convenience sample of predominantly White British parents limiting the generalisability of findings. Future research needs to compare levels of positive mental health between larger samples of mothers and fathers, ideally drawing on population‐representative designs. Further, all study measures were reported by parents, which may have led to social desirability bias, with parents reporting more favourable responses to avoid being stigmatised (Grimm 2010). Cridland et al. (2013) defined boundaries as hypothetical lines that divide a family and its surroundings. Parents of children with intellectual disability may be reluctant to share their true experiences with researchers outside their family boundaries, who may not fully understand their lived experiences. The selection of confounder variables for the models was restricted by the absence of theoretical models of positive well‐being, limited prior evidence in this area and data availability in the 1000 Families Study. The atheoretical selection of variables and the limited prior empirical evidence make it likely that theoretically important variables were missed. Future research exploring mother–father mental health differences should control for a broader set of likely confounders, in particular, psychological processes (e.g., locus of control, self‐efficacy and hope), which have been identified as important for subjective well‐being in disability studies (Boyraz and Sayger 2010; Shenaar‐Golan 2016).

In terms of practical implications, subject to replication in future studies, initial evidence of gender differences in life satisfaction could suggest that fathers, and men in general, may require additional support for achieving positive mental health. Workplaces and services should promote better work‐life balance for fathers to increase their ability to engage effectively with their children and partners, especially when their child has an intellectual disability. While this can be challenging to implement in practice, supporting the development of appropriate policy around extended parental leave and flexible working hours for fathers could be beneficial (e.g., Shared Parental Leave policy; UK Parliament 2023). Additionally, interventions focusing on interparental relationship quality may also improve parental positive mental health in families of children with intellectual disabilities.

## Conflicts of Interest

The authors declare no conflicts of interest.

## Data Availability

Data from this research study are not available for sharing due to ethical approval requirements. Researchers interested in collaboration should contact Professor Richard Hastings with their expression of interest.
